# Supplementary material for: Ecosystem health shapes viral ecology in peatland soils
Source: Nat Microbiol. 2025 Dec 10;11(1):142–54. doi: 10.1038/s41564-025-02199-x (PMC12768967; doi:10.1038/s41564-025-02199-x)
Supplement: Supplementary file 1 — Supplementary Methods, Results and References for Supplementary Information. [file 41564_2025_2199_MOESM1_ESM.pdf]

nature microbiology

Supplementary information https://doi.org/10.1038/s41564-025-02199-x

Ecosystem health shapes viral ecology in peatland soils

In the format provided by the authors and unedited

Supplementary Methods

Plotting

The map of Britain was generated in R with the packages maps[1] v3.4.2, mapdata[2] v2.3.1, and ggplot2[3] v3.5.1. The factoextra[4] package (v1.0.7) was used to extract PCA scores, loadings, and biplots, which were plotted using the "fviz_pca_biplot" function with 95% confidence ellipses around each Ecosystem Health Status (EHS) category. UpSet plots were generated with the R package ComplexUpset[5,6] v1.3.3. All other plots were generated in R using ggplot2 unless noted otherwise.

vMAG Genome Clustering with Soil Viral Genome Databases

The pairwise AAI measurements were used to compute genome-wide AAI for all possible viral genome comparisons using custom Python scripts (available at https://github.com/AnantharamanLab/UKPeatlandViruses). To form approximately genus-level viral genome clusters, we required genome pairs to have a minimum AAI of 40%, adhering to previously established benchmarks[7]. In addition to the 40% AAI threshold, genome pairs were required to share at least 16 protein sequences or have at least 20% of the genome with fewer genes shared with the other genome, consistent with past benchmarks for genus-level virus clustering[7]. "Shared" was defined as having a reported MMseqs2 alignment with at least 30% coverage. After applying these filters, the remaining pairwise comparisons were provided to MCL[8] v14-137 as an "abc" table and clustered with an inflation value of 2.0, which has previously been shown to be optimal for genus-level viral clusters[7]. The resulting MCL output table was used to assess the co-occurrence of viral genomes from multiple databases within the same genus-level cluster.

Statistical Analyses of Viral and Host Community Composition

To assess viral community composition and the factors that drive it, we first normalized the table of trimmed mean genome coverages for all species-representative vMAGs across all sample sites and EHS replicates by sequencing depth (in hundreds of millions of filtered reads per sample library). Since the Langwell site had nine restored peatland replicates representing soils with three different periods since restoration (Supplementary Table 1), genome abundance and genome coverage data from only the three replicates from soils with the longest restoration period (6 years before sampling) were retained to ensure consistency across sites for both community composition and downstream analyses. Viral and host genomes originally assembled from the removed replicates were retained, as species representatives assembled from them could still be present in other soils.

Using the genome breadth table, any viral species representative with a genome breadth <50% in a sample had its trimmed mean coverage set to zero to avoid false detection. The modified, normalized trimmed mean coverage table for all samples was then converted into a Bray-Curtis dissimilarity matrix in R using the vegan package[9] (v2.6.6.1). This matrix was subjected to principal coordinates analysis (PCoA) with the package ape[10] v5.8. We conducted an analysis of similarity (ANOSIM) on the same dissimilarity

matrix with the vegan package, using sample site as the grouping variable. Separate PCoA and ANOSIM analyses were also performed for each site, with EHS as the grouping variable. This process of normalization, modification of trimmed mean genome coverages, Bray-Curtis dissimilarity generation, PCoA, and ANOSIM was repeated for host species-representative genomes. The first axis from the virus PCoA was extracted and regressed against ecosystem health index (EHI)[11] with the function "geom_smooth" from the R package ggpubr[12] v0.6.0 using the method "lm." PCoA plots for viruses and hosts were generated using ggplot2.

To further investigate the drivers of viral community composition, we considered both biotic (host community composition) and abiotic factors (sample site, EHS, and EHI). Before running a Permutational Multivariate Analysis of Variance (PerMANOVA), we checked the beta-dispersion of the viral Bray-Curtis dissimilarity matrix with respect to sample site and EHS with a permutation test (n = 999 permutations) using the "betadisper" and "permutest" functions from the vegan package. Since the three EHS did not differ significantly in their average distance to the group centroid, but the seven sample sites did (see Supplementary Results), we performed the PerMANOVA holding sampling site constant by blocking by site, allowing us to control for site-level heterogeneity. We ran the PerMANOVA using the "adonis2" function from the vegan package (n = 9,999 permutations), with the Bray-Curtis dissimilarity matrix of viral communities across all sites as the response variable. Predictor variables, in order, included the first four PCoA axes of host community composition, followed by EHI and EHS. PerMANOVA P-values were adjusted using the Benjamini-Hochberg (BH) method.

To quantify the contributions of host community composition, sample site, and EHS to the variance in viral community composition, we conducted variance partitioning using the "varpart" function from the vegan package. The response variable was the virus Bray-Curtis dissimilarity matrix, with predictors being the host PCoA axes (X), sample site (X_1), and EHS (X_2). The statistical significance of variance partitioning results was assessed using Distance-based Redundancy Analysis (dbRDA) for sample site, EHS, and host community composition, separately. dbRDA was performed with the "dbrda" function from vegan, followed by Analysis of Variance (ANOVA) on each fitted dbRDA model. For all dbRDAs, the response variable was the virus Bray-Curtis dissimilarity matrix. In the sample site dbRDA, sample site was the predictor, with EHS and the first four host PCoA axes as conditioning terms to partition out variance from these factors. In the EHS dbRDA, EHS was the predictor, with sample site and host PCoA axes as conditioning terms. For the host community composition dbRDA, the first four host PCoA axes were predictors, with sample site and EHS as conditioning terms.

Host MAG Metabolic Function Predictions

To infer putative metabolic functions encoded by hosts, we used METABOLIC[13] v4.0, running the METABOLIC-C.pl program on all medium- and high-quality host MAGs with default parameters. We focused on eight metabolic functional categories relevant to peatland soil ecosystems–oxidative phosphorylation, methanogenesis, fermentation, carbohydrate degradation, aromatics degradation, assimilatory sulfate reduction, dissimilatory sulfate reduction, and thiosulfate oxidation–due to their recognized

ecological importance in soil carbon and sulfur cycling and their expected sensitivity to peatland disturbance and restoration[14–21]. The "KEGGModuleHit" results from METABOLIC-C were used to determine whether six of these functions were encoded by host MAGs. For oxidative phosphorylation, we considered the function to be present in a MAG if either of the KEGG[22] modules M00155 (cytochrome C oxidase, prokaryotes) or M00156 (cytochrome C oxidase, cbb3-type) was marked as present. For methanogenesis, the presence of any of the KEGG modules M00567 (hydrogenotrophic methanogenesis), M00357 (acetoclastic methanogenesis), M00356 (methylotrophic methanogenesis), or M00563 (methylotrophic methanogenesis from methylamine) indicated this function. For aromatics degradation, any of the following KEGG modules had to be present (see Supplementary Table 5 for descriptions): M00418, M00419, M00534, M00537, M00538, M00539, M00540, M00541, M00543, M00544, M00545, M00547, M00548, M00551, M00568, M00569, M00623, M00624, M00636, M00637, M00638, M00878, or M00915. Dissimilatory and assimilatory sulfate reduction functions were considered present if KEGG modules M00596 and M00176, respectively, were detected. For thiosulfate oxidation, the presence of module M00595 was required.

Since no KEGG modules are directly associated with fermentation or carbohydrate degradation, and since the latter function is typically encoded by individual enzymes[23], we used the "FunctionHit" results from METABOLIC-C to assess the presence or absence of these functions in each MAG. For fermentation, any of the six functions listed under the "fermentation" category in the METABOLIC-C results had to be present for the function to be considered encoded. Similarly, we checked the "complex carbon degradation" category to determine whether the carbohydrate degradation function was present in each MAG. Descriptions of the specific pathways in these two categories are provided in Supplementary Table 5.

Viral and Host Relative Abundance Across EHS Groups

To analyze changes in virus and host abundances across EHS with respect to host taxonomy and metabolic functions, we filtered the normalized, modified trimmed mean genome coverages for virus and host genomes (as used for PCoA above) to include only the differentially abundant viral/host genomes assigned to one of the three EHS groups. Additionally, to prevent skewing in abundance data due to non-detection, we included only genomes with trimmed mean genome coverage values >0, as the trimmed means were modified to set values to zero when a vMAG/MAG had a genome breadth <50%.

The filtered trimmed mean genome coverage data were converted into relative abundance values separately for viruses and hosts. These relative abundances were calculated across EHS groups, using the combined data from samples of all sites, to determine the overall relative abundance of viral and host genomes relative to other trend groups.

To analyze the composition of viruses and hosts within each trend group according to host class, relative abundances were recalculated from the filtered trimmed mean genome coverages. In this case, the abundances of hosts and their associated viruses were determined relative to other host classes within the same trend group. This

calculation was performed separately for each sample site, as well as for samples combined across all sites.

Since the eight metabolic functions of interest are not mutually exclusive (a host can encode multiple functions), relative abundances for hosts encoding each function and their associated viruses were calculated separately. Specifically, the relative abundance of hosts encoding a particular function and their viruses was determined relative to other hosts/viruses with the same function across different trend groups, rather than relative to other metabolic functions within the same trend group. To identify whether a host metabolic function was enriched in a particular EHS group, the virus and host relative abundance values for each trend group-metabolism combination were normalized by the previously calculated overall relative abundances of viruses and hosts in each trend group. This produced a relative abundance ratio for comparison. The associated reported changes in relative abundance ratios reflect aggregated populations at the species level rather than individual replicates, limiting the applicability of traditional statistical significance tests. These observations highlight biologically meaningful trends rather than statistically validated differences. All visualizations of relative abundance were generated using ggplot2.

Lysogenic Virus Abundance and Statistical Analysis of Active Viruses

To examine temperate (hereafter lysogenic) phage abundance across EHS, lysogenic viruses were first identified by filtering vMAGs in Supplementary Table 3 based on their lytic state as reported by ViWrap[24] (see github.com/AnantharamanLab/ViWrap#notes and github.com/AnantharamanLab/vRhyme#interpreting-vrhyme-binsvmags-), retaining those classified as “integrated prophage,” “lysogenic virus,” or “lysogenic scaffold.” Trimmed mean genome coverages of vMAGs in all samples were filtered to include only these identified lysogenic viruses and values >0. To account for differences in total viral load across samples, we normalized lysogenic virus abundance by dividing each lysogenic virus's abundance by the total virus abundance in the same sample. We then summarized the normalized lysogenic abundance per sample by calculating the mean, standard deviation, and standard error. All abundance values and summaries were computed at the sample level to avoid pseudoreplication. To test for differences in normalized lysogenic virus abundance across EHS, we used linear mixed-effects models (LMMs) with the “lmer” function from the R package lme4[25] v1.1.35.5, with EHS as a fixed effect and sample site as a random intercept to account for random variation across sites. Pairwise comparisons between EHS were computed using estimated marginal means with Benjamini-Hochberg adjustment.

We also modeled the relationship between normalized lysogenic virus abundance and the continuous EHI using an LMM, again with sample site as a random intercept. Model comparisons were performed using likelihood ratio tests to determine whether inclusion of the EHI or treatment categories improved model fit. The final selected model included the EHI as a fixed effect and site as a random intercept. Model diagnostics were performed to evaluate residual behavior, influential sites, and model robustness. Marginal and conditional R^2 values were calculated using the “r.squaredGLMM” function from the

R package MuMIn[26] v1.48.4 to quantify variance explained by fixed effects alone versus both fixed and random effects combined.

Finally, to explore virus replication activity, virus-to-host abundance ratios were calculated for all available pairs of individual vMAGs and host MAGs. A ratio of ≥10 was used to identify "active" viruses. All virus-host abundant ratios or hosts with taxonomic assignments at the family level were modeled using a linear mixed-effects model with the "lmer" function from the R package lme4[25], with virus-host ratio as the response variable and the interaction between EHS and host family as a fixed effect, accounting for variation between samples, sample sites, viruses, and host genomes by including each as a random intercept. Type II and type III ANOVAs were performed on the linear mixed-effects model to assess the significance of the fixed effects and their interactions.

vMAG Protein Functional Annotations

An HMMsearch[27] was performed on all vMAG-encoded amino acid sequences using profile HMMs from PHROGs[28] (release 2022-01-17x), KEGG KOfam[22] (March 2019 release), Pfam-A[29] v35.0, CAZy[23] (release 8062022) as provided by dbCAN3[30], and the custom-built set of metabolic HMMs used by METABOLIC[13]. Each database was searched separately. HMM searches were conducted using PyHMMER[27,31] v0.10.10 through custom Python scripts (available at https://github.com/AnantharamanLab/UKPeatlandViruses), enforcing a minimum alignment coverage of 50% to ensure that alignments were representative of whole proteins rather than individual domains. In cases where an amino acid sequence had more than one hit to profile HMMs within the same database, only the hit with the highest bitscore was retained.

Auxiliary Metabolic Gene (AMG) Prediction and Curation

To identify putative AMGs encoded by vMAGs, we employed a stringent approach that utilized functional annotations and genomic context statistics to avoid false-positive predictions, following community standards[32].

First, we identified proteins with metabolic functions by analyzing the functional annotations of the highest-scoring alignment for each protein. In cases where a protein only aligned to a profile HMM from a single database, that single annotation was considered. A protein was classified as metabolic if it met at least one of the following criteria: (1) the highest-scoring alignment was to a profile HMM in the METABOLIC custom HMM database, (2) the highest-scoring alignment was to a Carbohydrate-Active Enzyme (CAZyme) profile HMM, (3) the highest-scoring alignment was to a profile in the KEGG KOfam database and the protein was present in the list of AMG KOs used by the virus and AMG identification tool VIBRANT[33] (see github.com/AnantharamanLab/VIBRANT/blob/master/files/VIBRANT_AMGs.tsv), excluding KOs in the category "nucleotide metabolism", or (4) the highest-scoring alignment was to a PHROG profile HMM (since PHROGs encompass putative AMGs as well as other proteins), and the protein also had an annotation matching a CAZyme,

METABOLIC, or KEGG KOfam profile HMM present in the list of VIBRANT AMG KOs, excluding those involved in nucleotide metabolism.

Next, we filtered the putative metabolic proteins to remove spurious predictions likely resulting from homology to proteins with non-metabolic functions[32,34]. A list of "false" AMG keywords was compiled (Supplementary Table 6) to identify potential misclassifications due to homology with proteins associated with lysis, genome replication, anti-host defense, genome degradation, transcriptional regulation, and capsid assembly (Supplementary Table 6). If a protein had an annotation to a profile HMM in KEGG KOfam, Pfam, CAZyme, METABOLIC database, or PHROGs containing any of these keywords, it was excluded from the list of putative AMGs. While we acknowledge that this stringent filtering may remove some *bona fide* AMGs in addition to misclassified ones, we adopted this conservative approach due to the limited representation of soil viral genomes and proteins in public databases and the generally poor understanding of their functional roles.

Finally, AMG predictions were further refined to include only AMGs unlikely to have originated from host genome contamination[32]. This was achieved by assigning V-scores and V_L-scores to AMGs, as described by Zhou et al. (2024). V-scores and V_L-scores are indicative of whether a protein is typically encoded by viruses or non-viral sources[35]. For an AMG to be considered valid, we required it to be flanked within 10 kb on both sides by another protein annotated to a profile HMM with a V-score of 10, signifying a strong viral association[35]. Additionally, the local genomic region encoding the putative AMG (10kb window) was required to have an overall average V_L-score of at least 3.15, ensuring the region overall is of viral origin[35].

Supplementary Results

Metagenome Assembly, Quality, and Metagenome-Assembled Genome (MAG) Generation

Metagenomic sequence read quality control suggested all 66 paired-read libraries were of high-quality, with >95% of pairs retained after filtering each library (Supplementary Table 1). We generated 22 metagenomes that were co-assembled from the filtered metagenomic sequence read libraries (Supplementary Table 1). Altogether, the 22 co-assemblies contained 606,380 contigs at least 5 kb in length, with an overall average length of 27.56 $\pm$ 5.62 kb. The average N50 and L50 across all co-assemblies were 2,974 and 66,150, respectively (Supplementary Table 1). Mapping filtered reads back to their assemblies revealed an overall read recruitment of 61.01% to all contigs (Supplementary Table 2), 59.80 $\pm$ 7.69% per co-assembly or 59.97 $\pm$ 9.25% per sample (Supplementary Table 2). Binning metagenomic contigs with MetaBAT2 generated an initial 3,098 bins, 141 $\pm$ 41 per co-assembly on average (Supplementary Table 7). Of these, a total of 935 bins were estimated to be medium- or high-quality MAGs. Taxonomic assignments by GTDB-tk revealed 73 Archaeal MAGs from 5 phyla and 862 Bacterial MAGs from 21 phyla among the medium- and high-quality MAGs. Clustering the 935 medium- and high-quality MAGs generated 459 species-level representative MAGs that were used for downstream beta-diversity and differential abundance analyses.

Host Community Composition

Using principal coordinates analysis (PCoA) of all sample sites combined, we found that geography was the primary driver of host community composition (Extended Data Figure 1A). Although the host communities of some samples from different sites were similar in composition (Extended Data Figure 1A), samples in the PCoA were generally grouped by their geographic origin (R = 0.679, P = 0.001, ANOSIM) (Extended Data Figure 1A). This was consistent with our analysis of viral community composition (Figure 2D). As with the viral communities, a strong separation of host communities by ecosystem health status (EHS) was seen when performing PCoA on host MAGs from each site, separately (Extended Data Figure 1B), with statistics supporting this (R >0.5, P <0.05, ANOSIM) in all sites except Stean, as was observed for the viral communities (Figure 2E). Overall, these suggest that geography and EHS significantly and strongly governed host community composition similarly as they did with virus community composition.

Host Genome Differential Abundance Across Ecosystem Health Statuses

Using DESeq2, 455 out of 459 host species representative genomes (99%) were differentially abundant across EHS in at least one sample (P < 0.05, FDR adjusted, likelihood-ratio test, see Source Data Extended Data Figure 4). Hierarchical clustering of the mean abundance z-scores of these 455 representative genomes across samples into EHS ‘trend groups’ revealed that 664 (40%) were enriched in natural soils, 440 (26%) in restored soils, and 560 (34%) in damaged soils (Extended Data Figure 3). The sum of host species representatives across all trend groups exceeds the total number of unique representative genomes. This occurs because the same set of 445 differentially abundant representative genomes was used for hierarchical clustering across all sites (see Methods), allowing for one species representative to be enriched in one EHS at one sample site and represent a host of the same species enriched in a different EHS at another site. Across all sites, there was a greater proportion of natural-enriched hosts (39%) than damaged-enriched viruses (34%) and restored-enriched viruses (27%) (Figure 3A).

Effects of Host Community Composition on Viral Community Composition

Viruses, as obligate parasites, are closely tied to their hosts. To explore factors shaping viral communities, we analyzed how sample site and ecosystem health affect viral composition alongside host communities. Multivariate dispersion differed among sites (P = 0.001, permutation test, n = 999 permutations) while dispersion among treatments remained homogeneous (P = 0.168), prompting us to treat sampling site as a blocking factor in a subsequent PerMANOVA on community composition. After controlling for variation due to sampling site, host-community composition (host PCoA axes 1-4) explained the largest share of within-site variation (cumulative marginal R^2 = 0.38, P = 0.005 for each axis, PerMANOVA, n = 9,999 permutations; Supplementary Table 4). Ecosystem health also influenced community composition but to a lesser extent: the categorical EHS accounted for 5.1% of viral Bray-Curtis variation (F = 2.97, P = 0.001), whereas EHI explained 2.9% (F = 3.35, P = 0.0045). Variance partitioning of the same model corroborated these results. The pure fractions showed that host composition

contributed an adjusted R^2 = 0.187 to viral community structure, followed by sample site (adj. R^2 = 0.156; Supplementary Table 4). EHS also contributed an adjusted R^2 = 0.025 to viral community structure, and ecosystem health index (EHI) contributed an adjusted R^2 = 0.005 (Supplementary Table 4). Partial distance-based redundancy analysis (db-RDA) further showed that these were significant (host axes: F = 7.6, P = 0.001; sample site: F = 4.8, P = 0.001; EHS: F = 2.7, P = 0.001; EHI: F = 1.6, P = 0.039; Supplementary Table 4). In summary, although viral community composition is intricately linked to host community structure, our findings show that geography and ecosystem health (see *Results*) also impact viral communities in ways that are separate from their hosts.

Virus Species Representative Totals Across Trend Groups

Using DESeq2, we found that 1,448 out of 1,548 viral species representative genomes (94%) showed significant changes in abundance across EHS in at least one site (P <0.05, FDR adjusted, likelihood-ratio test, see Source Data Extended Data Figure 3 for individual statistics). Hierarchical clustering of the mean abundance z-scores of these 1,448 representative genomes across samples into EHS 'trend groups' revealed that 965 (35%) were enriched in natural soils, 895 (32%) in restored soils, and 897 (33%) in damaged soils (Extended Data Figure 3).

It is important to note that the sum of virus species representatives across all trend groups in Extended Data Figure 3 and its Source Data exceeds the total number of unique representative genomes. This occurs because the same set of 1,448 differentially abundant representative genomes was used for hierarchical clustering across all sites (more details in Methods). This approach allowed for one virus species representative to be enriched in one EHS at one sample site and also represent a virus of the same species enriched in a different EHS at another site.

Note that endemicity (presence exclusively in one EHS across all sites), as determined by our presence/absence analysis (see main text), and differential abundance (significant abundance variation between EHS within individual sites) represent complementary but distinct measures of viral distribution. Viruses endemic to a single EHS inherently differ in abundance from EHS where they are absent, while viruses present in multiple EHS can still show significant abundance shifts, contributing to the higher proportion (94%) of differentially abundant viruses reported. Additionally, virus species representatives are clusters dereplicated at 95% identity, allowing for variable differential abundance patterns across sites.

Viral and Host Dynamics Across Key Biogeochemical Functions in Peatlands

The eight metabolic functions we evaluated were oxidative phosphorylation, methanogenesis, fermentation, carbohydrate degradation, aromatics degradation, and sulfur cycling (including assimilatory and dissimilatory sulfate reduction, as well as thiosulfate oxidation) (Supplementary Table 5).

For oxidative phosphorylation, the relative abundance of viruses increased from natural (1.00) to restored (1.12 +11%), then decreased from restored to damaged (0.89, -25%),

while the host abundance decreased from natural (1.08) to restored (0.82, -32%), and slightly increased from natural to damaged (1.06, -1.85%) (Figure 3C).

In methanogenesis, viruses were only detected in restored soils, where they had a much higher relative abundance ratio (2.8, n = 1 virus genome) compared to its predicted host species representatives (1.8, n = 2 host genomes), suggesting an enrichment of viruses infecting methanogens in restored peatland soils (Figure 3C). Although this observation involves only a single viral species representative genome and two host representative genomes, each represents an aggregated species-level population across multiple samples and replicates. Thus, the n = 1 virus or n = 2 hosts here should not be taken as literally a single individual, but rather as capturing an entire lineage present in the community.

For fermentation, viral abundance increased slightly from natural (1.07) to restored (1.08, +1.7%), and decreased in damaged soils (0.87, -22%). Host abundance showed a decrease from natural (1.13) to restored (0.99, -14%) and further decreased in damaged soils (0.86, -32%) (Figure 3C).

Carbohydrate degradation saw minor changes, with viral abundance stable from natural (1.05) to restored (1.05), and a slight decrease in damaged soils (0.91, -15%). Host abundance was similarly stable from natural (1.03) to restored (0.95, -9%), with minimal change in damaged soils (1.01) (Figure 3C).

In aromatics degradation, viral abundance increased from natural (0.87) to damaged (1.12, +22%), and similarly, host abundance increased from natural (0.68) to damaged (1.17, +44%), suggesting a synchronized enrichment of viruses and hosts involved in aromatic degradation processes (Figure 3C).

For assimilatory sulfate reduction, viral abundance decreased significantly from natural (1.19) to restored (0.78, -52%), with host abundance showing a drastic decline from natural (1.07) to restored (0.41, -159%). From restored to damaged soils, viral abundance increased by 25% (0.78 to 1.04), while host abundance showed a major increase from 0.41 to 1.38 (70%, Figure 3C).

For dissimilatory sulfate reduction, viral abundance decreased by 36% (from 1.15 to 0.85) from natural to restored soils, with a moderate recovery (+17%) in damaged soils (to 1.02). Host abundance showed a sharp decline across all transitions: natural (1.32) to restored (0.90, -48%) and further to damaged soils (0.72, -25%) (Figure 3C).

In thiosulfate oxidation, viral abundance increased dramatically by 72% (from 0.43 to 1.57) between restored and damaged soils, following a large decrease from natural to restored (-111%). Host abundance showed a significant decline from natural (1.71) to restored soils (0.57, -199%) but remained relatively stable between restored and damaged soils (-5%, from 0.57 to 0.54) (Figure 3C).

Virus-Host Infection Dynamics Change with Ecosystem Health

The variation in virus-host abundance slopes across EHS highlights how restoration efforts influence virus-host dynamics (Figure 5A). In *Desulfobacterota*, strong and stable correlations are observed in both natural (m = 0.620, R^2 = 0.96, P = 5.05e-9, BH-adjusted) and restored soils (m = 0.920, R^2 = 0.95, P = 5.05e-9, BH-adjusted), indicating consistent virus-host interactions in these environments (Figure 5A). However, in damaged soils, the relationship becomes insignificant (m = 0.078, $R^2 \approx 0$, P = 0.856, BH-adjusted), suggesting a disruption in virus-host dynamics under degraded conditions. In contrast, *Desulfobacterota_B* shows a marginally significant relationship in natural soils (m = 0.672, R^2 = 0.88, P = 0.066, BH-adjusted), but much stronger correlations in restored (slope = 0.682, R^2 = 0.94, P = 1.4e-6) and damaged soils (m = 0.680, R^2 = 0.77, P = 6.6e-05, BH-adjusted), indicating that virus-host dynamics within this phylum are more robust in disturbed environments. *Actinomycetota* exhibits significant but more varied slopes, with a moderate correlation in natural soils (m = 0.404, R^2 = 0.84, P = 3.01e-7, BH-adjusted), weaker relationships in restored (m = 0.198, R^2 = 0.61, P = 3.25e-4, BH-adjusted) and damaged soils (m = 0.305, R^2 = 0.54, P = 4.02e-4, BH-adjusted), suggesting shifting interactions across EHS. In *Pseudomonadota*, the correlation is weak but significant in natural soils (m = 0.247, R^2 = 0.27, P = 0.0412, BH-adjusted), stronger in restored soils (m = 0.621, R^2 = 0.90, P = 2.56e-7, BH-adjusted), and remains significant in damaged soils (m = 0.436, R^2 = 0.92, P = 7.01e-8, BH-adjusted), indicating that this phylum may be more sensitive to environmental conditions, leading to less predictable virus-host dynamics. Overall, these results suggest that EHS has a differential impact on virus-host interactions, with some phyla showing stable ‘piggyback-the-winner’ relationships across EHS, while others exhibit more complex, environment-dependent dynamics but still indicative of non-lytic infection. However, caution in interpretation is warranted due to potential biases in viral detection from microbial metagenomes, which may preferentially represent intracellular viruses.

To further explore the impact of EHS on virus lifestyle, we modeled virus/host ratios as a function of EHS and host family, accounting for variation between samples, sample sites, host genomes, and viral genomes. The host family of viruses significantly affected virus/host ratios (P <0.05), demonstrating that different host families exhibit distinct virus-host interaction dynamics across all EHS. Ecosystem health alone did not have a significant effect (P >0.05); however, the model revealed a highly significant interaction between EHS and host family (Type II ANOVA, P <0.0001, Supplementary Table 8), indicating an effect of EHS on virus/host ratios that varies considerably across host families. When accounting for this variation due to host family across EHS, it became clear that EHS had a highly significant effect on virus/host ratios (Type III ANOVA, P <0.001, Supplementary Table 8). Overall, these results reinforce our findings that environmental changes in peatland soils reshape virus-host interactions in a lineage-specific manner.

References

1. Becker, R. A., Wilks, A. R. & Brownrigg, R. maps: Draw Maps. Preprint at https://CRAN.R-project.org/package=maps (2023).
2. Becker, R. A., Wilks, A. R. & Brownrigg, R. mapdata: Extra Map Databases. Preprint at https://doi.org/10.32614/CRAN.package.mapdata (2022).

3. Wickham, H. *Ggplot2: Elegant Graphics for Data Analysis*. (Springer-Verlag New York, 2016).
4. Kassambara, A. & Mundt, F. factoextra: Extract and Visualize the Results of Multivariate Data Analyses. Preprint at https://github.com/kassambara/factoextra (2020).
5. Krassowski, M. ComplexUpset. Preprint at https://doi.org/10.5281/zenodo.3700590 (2020).
6. Lex, A., Gehlenborg, N., Vuillemot, H. S. and R. & Pfister, H. UpSet: Visualization of Intersecting Sets,. *IEEE Trans Vis Comput Graph* **20**, 1983–1992 (2014).
7. Nayfach, S. *et al.* Metagenomic compendium of 189,680 DNA viruses from the human gut microbiome. *Nat Microbiol* **6**, 960–970 (2021).
8. Van Dongen, S. Graph Clustering Via a Discrete Uncoupling Process. *SIAM Journal on Matrix Analysis and Applications* **30**, 121–141 (2008).
9. Oksanen J *et al.* vegan: Community Ecology Package. Preprint at (2018).
10. Paradis, E. & Schliep, K. ape 5.0: an environment for modern phylogenetics and evolutionary analyses in R. *Bioinformatics* **35**, 526–528 (2019).
11. Pallier, W. *et al.* Recovery of microbial ecophysiology and carbon accrual functions in peatlands under restoration. *bioRxiv* 2025.08.01.668219 (2025) doi:10.1101/2025.08.01.668219.
12. Kassambara, A. ggpubr: 'ggplot2' Based Publication Ready Plots. Preprint at https://rpkgs.datanovia.com/ggpubr/ (2023).
13. Zhou, Z. *et al.* METABOLIC: high-throughput profiling of microbial genomes for functional traits, metabolism, biogeochemistry, and community-scale functional networks. *Microbiome* **10**, 33 (2022).
14. Robinson, C. H. *et al.* Aspects of Microbial Communities in Peatland Carbon Cycling Under Changing Climate and Land Use Pressures. *Mires and Peat* **29**, 2 (2023).
15. Pester, M. Sulfate-reducing microorganisms in wetlands – fameless actors in carbon cycling and climate change. *Front Microbiol* **3**, (2012).
16. Fenner, N. & Freeman, C. Woody litter protects peat carbon stocks during drought. *Nat Clim Chang* **10**, 363–369 (2020).
17. Kitson, E. & Bell, N. G. A. The Response of Microbial Communities to Peatland Drainage and Rewetting. A Review. *Front Microbiol* **11**, (2020).
18. Dalcin Martins, P. *et al.* Abundant carbon substrates drive extremely high sulfate reduction rates and methane fluxes in Prairie Pothole Wetlands. *Glob Chang Biol* **23**, 3107–3120 (2017).
19. McGivern, B. B. *et al.* Microbial polyphenol metabolism is part of the thawing permafrost carbon cycle. *Nat Microbiol* **9**, 1454–1466 (2024).
20. Brooks Avery, G., Shannon, R. D., White, J. R., Martens, C. S. & Alperin, M. J. Controls on methane production in a tidal freshwater estuary and a peatland: methane production via acetate fermentation and CO2 reduction. *Biogeochemistry* **62**, 19–37 (2003).
21. Richy, E. *et al.* How microbial communities shape peatland carbon dynamics: New insights and implications. *Soil Biol Biochem* **191**, 109345 (2024).
22. Kanehisa, M., Sato, Y., Kawashima, M., Furumichi, M. & Tanabe, M. KEGG as a reference resource for gene and protein annotation. *Nucleic Acids Res* **44**, D457–D462 (2016).
23. Cantarel, B. L. *et al.* The Carbohydrate-Active EnZymes database (CAZy): an expert resource for Glycogenomics. *Nucleic Acids Res* **37**, D233–D238 (2009).
24. Zhou, Z., Martin, C., Kosmopoulos, J. C. & Anantharaman, K. ViWrap: A modular pipeline to identify, bin, classify, and predict viral–host relationships for viruses from metagenomes. *iMeta* https://doi.org/10.1002/imt2.118 (2023) doi:10.1002/imt2.118.
25. Bates, D., Mächler, M., Bolker, B. M. & Walker, S. C. Fitting linear mixed-effects models using lme4. *J Stat Softw* **67**, (2015).

26. Bartoń, K. MuMIn: Multi-Model Inference. Preprint at https://CRAN.R-project.org/package=MuMIn (2024).
27. Eddy, S. R. Accelerated Profile HMM Searches. *PLoS Comput Biol* **7**, e1002195 (2011).
28. Terzian, P. *et al.* PHROG: families of prokaryotic virus proteins clustered using remote homology. *NAR Genom Bioinform* **3**, lqab067 (2021).
29. Mistry, J. *et al.* Pfam: The protein families database in 2021. *Nucleic Acids Res* **49**, D412–D419 (2021).
30. Zheng, J. *et al.* dbCAN3: automated carbohydrate-active enzyme and substrate annotation. *Nucleic Acids Res* **51**, W115–W121 (2023).
31. Larralde, M. & Zeller, G. PyHMMER: a Python library binding to HMMER for efficient sequence analysis. *Bioinformatics* **39**, (2023).
32. Pratama, A. A. *et al.* Expanding standards in viromics: in silico evaluation of dsDNA viral genome identification, classification, and auxiliary metabolic gene curation. *PeerJ* **9**, e11447 (2021).
33. Kieft, K., Zhou, Z. & Anantharaman, K. VIBRANT: automated recovery, annotation and curation of microbial viruses, and evaluation of viral community function from genomic sequences. *Microbiome* **8**, 90 (2020).
34. ter Horst, A. M. *et al.* Minnesota peat viromes reveal terrestrial and aquatic niche partitioning for local and global viral populations. *Microbiome* **9**, (2021).
35. Zhou, K., Kosmopoulos, J. C., Colón, E. D., Badciong, P. J. & Anantharaman, K. V- and VL-Scores Uncover Viral Signatures and Origins of Protein Families. *bioRxiv* 2024.10.24.619987 (2024) doi:10.1101/2024.10.24.619987.
