# Supplementary material for: Ecosystem health shapes viral ecology in peatland soils
Source: Nat Microbiol. 2025 Dec 10;11(1):142–54. doi: 10.1038/s41564-025-02199-x (PMC12768967; doi:10.1038/s41564-025-02199-x)
Supplement: Supplementary file 2 — Reporting Summary [file 41564_2025_2199_MOESM2_ESM.pdf]

nature portfolio

Corresponding author(s): Ashish A. Malik & Karthik Anantharaman

Last updated by author(s): Sep 29, 2025

Reporting Summary

Nature Portfolio wishes to improve the reproducibility of the work that we publish. This form provides structure for consistency and transparency in reporting. For further information on Nature Portfolio policies, see our Editorial Policies and the Editorial Policy Checklist.

Statistics

For all statistical analyses, confirm that the following items are present in the figure legend, table legend, main text, or Methods section.

n/a	Confirmed	
☐	☒	The exact sample size (*n*) for each experimental group/condition, given as a discrete number and unit of measurement
☐	☒	A statement on whether measurements were taken from distinct samples or whether the same sample was measured repeatedly
☐	☒	The statistical test(s) used AND whether they are one- or two-sided<br>*Only common tests should be described solely by name; describe more complex techniques in the Methods section.*
☐	☒	A description of all covariates tested
☐	☒	A description of any assumptions or corrections, such as tests of normality and adjustment for multiple comparisons
☐	☒	A full description of the statistical parameters including central tendency (e.g. means) or other basic estimates (e.g. regression coefficient) AND variation (e.g. standard deviation) or associated estimates of uncertainty (e.g. confidence intervals)
☐	☒	For null hypothesis testing, the test statistic (e.g. *F*, *t*, *r*) with confidence intervals, effect sizes, degrees of freedom and *P* value noted<br>*Give P values as exact values whenever suitable.*
☒	☐	For Bayesian analysis, information on the choice of priors and Markov chain Monte Carlo settings
☐	☒	For hierarchical and complex designs, identification of the appropriate level for tests and full reporting of outcomes
☒	☐	Estimates of effect sizes (e.g. Cohen's *d*, Pearson's *r*), indicating how they were calculated

Our web collection on statistics for biologists contains articles on many of the points above.

Software and code

Policy information about availability of computer code

Data collection	All scripts for data processing and visualization are available via GitHub at https://github.com/AnantharamanLab/UKPeatlandViruses. The software used for analyses, and their versions, are as follows: Anvi'o v8, ape v5.8 (R package), Bowtie2 v2.4.5, Bowtie2 v2.5.1, CheckM v1.2.2, ComplexUpset v1.3.3 (R package), CoverM v0.6.1, DESeq2 v1.44.0 (R package), dRep v3.5.0, factoextra v1.0.7 (R package), GeNomad v1.7.4, ggplot2 v3.5.1 (R package), ggpubr v0.6.0 (R package), GTDB-Tk v2.3.2, Illumina-utils v2.13, iPHoP v1.3.3, lme4 v1.1.35.5 (R package), mapdata v2.3.1 (R package), maps v3.4.2 (R package), MCL v14-137, MEGAHIT v1.2.9, MetaBAT 2 v2.15, METABOLIC v4, metaQUAST v5.2.0, MMseqs2 v15.6f452, MuMIn v1.48.4 (R package), pyHMMER v0.10.10 (Python package), pyrodigal-gv v0.3.1 (Python package), Python v3.10.11, R v4.4.0, SAMtools v1.17, skani v0.2.1, vegan v2.6.6.1 (R package), ViWrap v1.3.0, vRhyme v1.1.0
Data analysis	All scripts for data processing and visualization are available via GitHub at https://github.com/AnantharamanLab/UKPeatlandViruses. The software used for analyses, and their versions, are as follows: Anvi'o v8, ape v5.8 (R package), Bowtie2 v2.4.5, Bowtie2 v2.5.1, CheckM v1.2.2, ComplexUpset v1.3.3 (R package), CoverM v0.6.1, DESeq2 v1.44.0 (R package), dRep v3.5.0, factoextra v1.0.7 (R package), GeNomad v1.7.4, ggplot2 v3.5.1 (R package), ggpubr v0.6.0 (R package), GTDB-Tk v2.3.2, Illumina-utils v2.13, iPHoP v1.3.3, lme4 v1.1.35.5 (R package), mapdata v2.3.1 (R package), maps v3.4.2 (R package), MCL v14-137, MEGAHIT v1.2.9, MetaBAT 2 v2.15, METABOLIC v4, metaQUAST v5.2.0, MMseqs2 v15.6f452, MuMIn v1.48.4 (R package), pyHMMER v0.10.10 (Python package), pyrodigal-gv v0.3.1 (Python package), Python v3.10.11, R v4.4.0, SAMtools v1.17, skani v0.2.1, vegan v2.6.6.1 (R package), ViWrap v1.3.0, vRhyme v1.1.0

For manuscripts utilizing custom algorithms or software that are central to the research but not yet described in published literature, software must be made available to editors and reviewers. We strongly encourage code deposition in a community repository (e.g. GitHub). See the Nature Portfolio guidelines for submitting code & software for further information.

Data

Policy information about availability of data

All manuscripts must include a data availability statement. This statement should provide the following information, where applicable:

- Accession codes, unique identifiers, or web links for publicly available datasets
- A description of any restrictions on data availability
- For clinical datasets or third party data, please ensure that the statement adheres to our policy

All raw sequencing data are publicly available in the NCBI Short Read Archive under BioProject accession PRJNA1203648. Whole assembled metagenomic contigs as well as high-quality prokaryotic metagenome-assembled genomes are available at the NCBI WGS using the same BioProject accession. All viral metagenome-assembled genomes and prokaryotic metagenome-assembled genomes (medium and high quality) are publicly available on figshare under the DOI 10.6084/m9.figshare.28143446. Source data are provided with this paper.

Research involving human participants, their data, or biological material

Policy information about studies with human participants or human data. See also policy information about sex, gender (identity/presentation), and sexual orientation and race, ethnicity and racism.

Reporting on sex and gender	*Use the terms sex (biological attribute) and gender (shaped by social and cultural circumstances) carefully in order to avoid confusing both terms. Indicate if findings apply to only one sex or gender; describe whether sex and gender were considered in study design; whether sex and/or gender was determined based on self-reporting or assigned and methods used. Provide in the source data disaggregated sex and gender data, where this information has been collected, and if consent has been obtained for sharing of individual-level data; provide overall numbers in this Reporting Summary. Please state if this information has not been collected. Report sex- and gender-based analyses where performed, justify reasons for lack of sex- and gender-based analysis.*
Reporting on race, ethnicity, or other socially relevant groupings	*Please specify the socially constructed or socially relevant categorization variable(s) used in your manuscript and explain why they were used. Please note that such variables should not be used as proxies for other socially constructed/relevant variables (for example, race or ethnicity should not be used as a proxy for socioeconomic status). Provide clear definitions of the relevant terms used, how they were provided (by the participants/respondents, the researchers, or third parties), and the method(s) used to classify people into the different categories (e.g. self-report, census or administrative data, social media data, etc.) Please provide details about how you controlled for confounding variables in your analyses.*
Population characteristics	*Describe the covariate-relevant population characteristics of the human research participants (e.g. age, genotypic information, past and current diagnosis and treatment categories). If you filled out the behavioural & social sciences study design questions and have nothing to add here, write "See above."*
Recruitment	*Describe how participants were recruited. Outline any potential self-selection bias or other biases that may be present and how these are likely to impact results.*
Ethics oversight	*Identify the organization(s) that approved the study protocol.*

Note that full information on the approval of the study protocol must also be provided in the manuscript.

Field-specific reporting

Please select the one below that is the best fit for your research. If you are not sure, read the appropriate sections before making your selection.

☐ Life sciences ☐ Behavioural & social sciences ☒ Ecological, evolutionary & environmental sciences

For a reference copy of the document with all sections, see nature.com/documents/nr-reporting-summary-flat.pdf

Ecological, evolutionary & environmental sciences study design

All studies must disclose on these points even when the disclosure is negative.

Study description	Total metagenome sequencing of 66 soil samples collected from seven upland peatland sites across Britain. Samples represent a gradient of ecosystem health statuses: near-natural benchmark, damaged, and restored, in close proximity to each other (three replicates per ecosystem health status).
Research sample	Soil samples were collected from seven upland peatland sites across Britain across a gradient of climatic conditions. At each site, we sampled three areas representing near-natural benchmark, damaged, and restored ecosystem health statuses in close proximity to each other (three replicates per ecosystem health status).
Sampling strategy	Triplicate samples were collected at each available ecosystem health level within every site to ensure sufficient statistical power for analyses and to examine both across-site and within-site variation.

Data collection	Soil samples were collected using a borer in triplicate across a 5-meter transect from the top 10cm layer of soil for each site x ecosystem health level combination.
Timing and spatial scale	Samples were collected at a single time point for each site, sampling sites between May and October of 2021. Seven upland peatland sites were sampled across Scotland, England, and Wales , chosen for their internal variation in ecosystem health levels.
Data exclusions	Since the Langwell site had nine restored peatland replicates (instead of three, as the other sites had) representing soils with three different periods since restoration, data from only the three replicates from soils with the longest restoration period were retained to ensure consistency across sites for both community composition and downstream analyses
Reproducibility	Triplicate samples were collected along a 5-meter transect at each ecosystem health level within every sampling site to ensure replication and reproducibility.
Randomization	Samples were grouped into triplicates representing the same sample site and ecosystem health levels. Covariance that may have been introduced by this grouping was accounted for in statistical analyses.
Blinding	Blinding is not relevant to this study since it involved sampling of environmental soil samples and sequencing their total community metagenomes.

Did the study involve field work? ☒ Yes ☐ No

Field work, collection and transport

Field conditions	Balmoral mean annual precipitation 1412mm mean annual temperature 5.5 C, Bowness mean annual precipitation 953mm mean annual temperature 9.6 C, Crocach mean annual precipitation 1258mm mean annual temperature 7.1 C, Langwell mean annual precipitation 1223mm mean annual temperature 7 C, Migneint mean annual precipitation 2181mm mean annual temperature 8 C, Moor House mean annual precipitation 1699mm mean annual temperature 8 C, Stean mean annual precipitation 1229mm mean annual temperature 8 C.
Location	Balmoral: 695 m elevation United Kingdom (Scotland) 56.92341 N 3.67514 W, Bowness: 66 m elevation United Kingdom (England) 54.93297 N 3.23945 W, Crocach: 189 m elevation United Kingdom (Scotland) 58.39304 N 4.00182 W, Migneint: 453 m elevation United Kingdom (Wales) 52.96932 N 3.81616 W, Moor House: 571 m elevation United Kingdom (England) 54.69457 N 2.37661 W, Stean: 530 m elevation United Kingdom (England) 54.13559 N 1.92875 W
Access & import/export	*Describe the efforts you have made to access habitats and to collect and import/export your samples in a responsible manner and in compliance with local, national and international laws, noting any permits that were obtained (give the name of the issuing authority, the date of issue, and any identifying information).*
Disturbance	*Describe any disturbance caused by the study and how it was minimized.*

Reporting for specific materials, systems and methods

We require information from authors about some types of materials, experimental systems and methods used in many studies. Here, indicate whether each material, system or method listed is relevant to your study. If you are not sure if a list item applies to your research, read the appropriate section before selecting a response.

Materials & experimental systems

n/a	Involved in the study	
☒	☐	Antibodies
☒	☐	Eukaryotic cell lines
☒	☐	Palaeontology and archaeology
☒	☐	Animals and other organisms
☒	☐	Clinical data
☒	☐	Dual use research of concern
☒	☐	Plants

Methods

n/a	Involved in the study	
☒	☐	ChIP-seq
☒	☐	Flow cytometry
☒	☐	MRI-based neuroimaging

Plants

Seed stocks	*Report on the source of all seed stocks or other plant material used. If applicable, state the seed stock centre and catalogue number. If plant specimens were collected from the field, describe the collection location, date and sampling procedures.*
Novel plant genotypes	*Describe the methods by which all novel plant genotypes were produced. This includes those generated by transgenic approaches, gene editing, chemical/radiation-based mutagenesis and hybridization. For transgenic lines, describe the transformation method, the number of independent lines analyzed and the generation upon which experiments were performed. For gene-edited lines, describe the editor used, the endogenous sequence targeted for editing, the targeting guide RNA sequence (if applicable) and how the editor was applied.*
Authentication	*Describe any authentication procedures for each seed stock used or novel genotype generated. Describe any experiments used to assess the effect of a mutation and, where applicable, how potential secondary effects (e.g. second site T-DNA insertions, mosiacism, off-target gene editing) were examined.*
